# Five‐year survival with nivolumab in previously untreated Japanese patients with advanced or recurrent malignant melanoma

**DOI:** 10.1111/1346-8138.15804

**Published:** 2021-03-14

**Authors:** Hisashi Uhara, Yoshio Kiyohara, Jiro Uehara, Yasuhiro Fujisawa, Tatsuya Takenouchi, Masaki Otsuka, Hiroshi Uchi, Satoshi Fukushima, Hironobu Minami, Masahiro Hatsumichi, Naoya Yamazaki

**Affiliations:** ^1^ Department of Dermatology Sapporo Medical University School of Medicine Sapporo Japan; ^2^ Dermatology Division Shizuoka Cancer Center Hospital Shizuoka Japan; ^3^ Department of Dermatologic Oncology Tokyo Metropolitan Cancer and Infectious Diseases Center Komagome Hospital Tokyo Japan; ^4^ Department of Dermatology University of Tsukuba Hospital Ibaraki Japan; ^5^ Department of Dermatology Niigata Cancer Center Hospital Niigata Japan; ^6^ Department of Dermato‐Oncology National Hospital Organization Kyushu Cancer Center Fukuoka Japan; ^7^ Department of Dermatology and Plastic Surgery Faculty of Life Sciences Kumamoto University Kumamoto Japan; ^8^ Department of Medical Oncology/Hematology Kobe University Graduate School of Medicine Hyogo Japan; ^9^ Ono Pharmaceutical Co., Ltd. Osaka Japan; ^10^ Department of Dermatologic Oncology National Cancer Center Hospital Tokyo Japan

**Keywords:** Japan, long‐term survivors, melanoma, nivolumab, survival

## Abstract

We report the 5‐year follow‐up results from a single‐arm, open‐label, multicenter phase II study (ONO‐4538‐08) conducted in Japan. Twenty‐four patients with treatment‐naïve, recurrent, or unresectable stage III/IV malignant melanoma received 3 mg/kg nivolumab every 2 weeks until progressive disease or unacceptable toxicity occurred. The 5‐year overall survival (OS) rate was 26.1%. Five years after the start of nivolumab treatment, there were six survivors. The 5‐year OS rate was 66.7% for patients with a superficial spreading type, 14.3% for acral lentiginous type, and 16.7% for mucosal type. The 5‐year progression‐free survival rate was 17.2%. No new cases of partial response or complete response were observed after 3 years, and overall response and disease control rates were similar to those reported at 3 years. The treatment‐related adverse events reported between the 3‐ and 5‐year follow‐up periods were anemia (grade 2), white blood cell count decrease (grade 2), and psoriasiform dermatitis (grade 2) in one patient each. No new grade 3 or higher treatment‐related adverse events occurred in this period. In conclusion, first‐line treatment with nivolumab in Japanese patients with unresectable or metastatic melanoma resulted in confirmed long‐term survival. No new safety signals were reported in the studied population.

## 1 INTRODUCTION

Advanced malignant melanoma patients have an extremely poor prognosis. Among Japanese patients with advanced malignant melanoma (disease stage classified according to the Cancer Staging Manual, 7th Edition,^1^ of the American Joint Committee on Cancer), the 5‐year disease‐specific survival rate has been reported as 75.0% for stage IIIA, 61.3% for stage IIIB, 41.7% for stage IIIC, and 17.7% for stage IV.^2^, ^3^ It should be noted that these survival estimates were calculated between 2005 and 2017, which means that these therapeutic efficacy assessments were made before the introduction of immune checkpoint inhibitors.

The anti‐programmed death (PD)‐1 monoclonal antibody nivolumab was first approved in 2014 as a treatment for previously treated unresectable/recurrent malignant melanoma.^3^ Prior to that, dacarbazine was the standard treatment for melanoma, and nivolumab provided an important treatment option. After nivolumab was granted initial approval in 2014, further approval of nivolumab as first‐line therapy was expected. To this end, a single‐arm, open‐label, multicenter phase II study in previously untreated Japanese patients with advanced (stage III or IV) or recurrent malignant melanoma (ONO‐4538‐08) was conducted and showed favorable efficacy and safety results with nivolumab treatment.^4^, ^5^ Patients achieved an overall response rate (ORR) of 34.8% and an overall survival (OS) rate of 56.5% at 18 months. Based on these results, nivolumab was approved as a first‐line treatment for advanced (stage III or IV) or recurrent malignant melanoma in Japan.

In CheckMate 066,^6^, ^7^, ^8^ an international phase III trial comparing nivolumab monotherapy with dacarbazine in patients with previously untreated *BRAF* wild‐type malignant melanoma, the 3‐ and 5‐year OS rates in the nivolumab monotherapy arm were 51.2% and 34%, respectively. In CheckMate 067,^9^, ^10^ an international phase III trial that involved three treatment arms (nivolumab monotherapy, ipilimumab monotherapy, and nivolumab + ipilimumab) in patients with previously untreated malignant melanoma, the 3‐ and 5‐year OS rates in the nivolumab monotherapy arm were 52% and 44%, respectively. However, predominant melanoma types are different between Japanese patients and non‐Japanese patients. Acral lentiginous melanoma predominates in Japan (~40%), whereas the superficial spreading type predominates in other countries.^2^ The proportions of acral lentiginous and mucosal melanoma types in Japan are higher than those reported in other countries^2^, ^11^, ^12^, ^13^, ^14^, ^15^ and, as these types are known to harbor tumor mutations far less frequently than the superficial spreading type,^15^ this may affect the effectiveness of nivolumab. Due to these differences, data confirming the long‐term efficacy of nivolumab in Japanese melanoma patients are needed.

We previously reported the 3‐year OS data in Japanese patients from the ONO‐4538‐08 study.^4^ Of 24 patients enrolled, there were 10 survivors, and the ORR and 3‐year OS rate were 34.8% and 43.5%, respectively. Here, we report the 5‐year follow‐up results, including stratification by melanoma type. The baseline characteristics of Japanese melanoma patients who were long‐term survivors are also reported.

## 2 METHODS

### 2.1 Study design

The study design of ONO‐4538‐08 has been described in a previous report.^5^ Briefly, the study was a single‐arm, open‐label, multicenter phase II study with three periods: screening, intervention, and post‐treatment follow‐up. Patients received nivolumab 3 mg/kg every 2 weeks as part of a 6‐week cycle until progressive disease or unacceptable toxicity. At the end of every cycle, patients were evaluated by computed tomography or magnetic resonance imaging to determine if they would continue treatment.

Treatment discontinuation criteria have been described previously.^4^, ^5^ Patients discontinued treatment if they presented complete response (CR), progressive disease (Response Evaluation Criteria in Solid Tumors [RECIST]), clinical signs of cancer progression, onset of grade 2 or more interstitial lung disease, grade 3 or more adverse events (AE), or grade 2 or more eye pain or reduced visual acuity grade that did not improve with topical treatment. If the treatment was discontinued, patients entered the follow‐up stage.

### 2.2 Patients

Patients enrolled in the study were Japanese individuals who had histopathologically confirmed unresectable stage III/IV or recurrent malignant melanoma based on the criteria from the Union for International Cancer Control TNM Classification of Malignant Tumors (7th edition). Additionally, enrolled patients had not previously received a systemic antineoplastic agent (e.g., chemotherapy, molecular targeted therapy, or immunotherapy), and had at least one measurable lesion by RECIST criteria (version 1.1). Previous pre‐ or postoperative adjuvant therapies for malignant melanoma were allowed if treatment had been concluded at least 6 weeks before enrollment, the condition of patients was stable, and all AE had reverted to the baseline conditions. Patients were excluded if they had a primary tumor in the esophagus or rectum or had multiple primary cancers.

### 2.3 Ethics

The institutional review board of each participating site approved the study protocol. The study conduct conformed to the provisions of the Declaration of Helsinki (as revised in Fortaleza, Brazil, October 2013) and the International Conference on Harmonisation Guideline for Good Clinical Practice. All participants provided written informed consent prior to initiation of study treatment. This study was registered at www.clinicaltrials.jp under the number JapicCTI‐142533.

### 2.4 End‐points

The primary end‐point was centrally assessed ORR; other efficacy end‐points were OS, progression‐free survival (PFS), best overall response (BOR), disease control rate (DCR), and percent change in the sum of diameters of the target lesions. Additionally, ORR, OS, PFS, and DCR were analyzed by presence of *BRAF V600* gene mutations in a subgroup analysis. Efficacy end‐points were also stratified by melanoma type. The cut‐off point for the sum of all diameters of the target lesions was determined using the quartile baseline tumor diameter (based on the first quartile [≤21.950 mm] and third quartile [>64.615 mm]).^4^ The safety end‐points were AE, treatment‐related AE, AE with potential immunological causes, and changes in vital signs, laboratory values, and 12‐lead electrocardiograms.

### 2.5 Statistical analysis

The sample size rationale and calculations, as well as the analysis populations, have been detailed previously.^5^ The planned overall sample size was at least 20 patients, of whom 14 were required to have *BRAF* wild‐type melanoma and six to have *BRAF* mutant melanomas. Summary statistics were used to analyze the demographic and baseline characteristics of patients. For primary and secondary efficacy end‐points, two‐sided 90% confidence intervals (CI) were calculated. The Kaplan–Meier method was used for survival analysis. A subgroup analysis was performed to determine the efficacy of nivolumab according to the sum of tumor diameters at baseline and melanoma type. The statistical software used was SAS version 9.3 or higher (SAS Institute).

## 3 RESULTS

### 3.1 Patient characteristics

All 24 patients enrolled in the phase II (ONO‐4538‐08) study^5^ were evaluated in this analysis. The date of enrollment of the last patient was 9 October 2014. The data cut‐off date was 31 October 2019. The baseline and demographic characteristics of patients have been described previously.^4^, ^5^ Acral lentiginous type was the most common melanoma type (29.2% [n = 7]), followed by mucosal (25.0% [n = 6]), superficial spreading (25.0% [n = 6]), nodular (4.2% [n = 1]), and unknown (16.7% [n = 4]). Of the 24 patients included in the primary analysis, one nodular type patient was found to have multiple tumors after the study was initiated. This patient was excluded from the efficacy analysis in line with the prespecified exclusion criteria.

### 3.2 Efficacy end‐points

**FIGURE 1:**
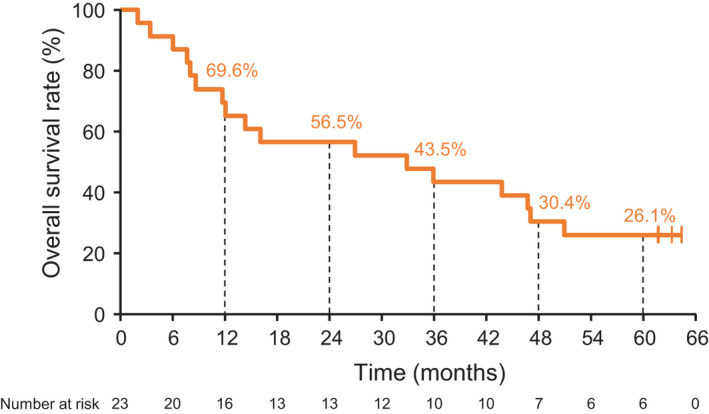
Kaplan–Meier curve of overall survival

**FIGURE 2:**
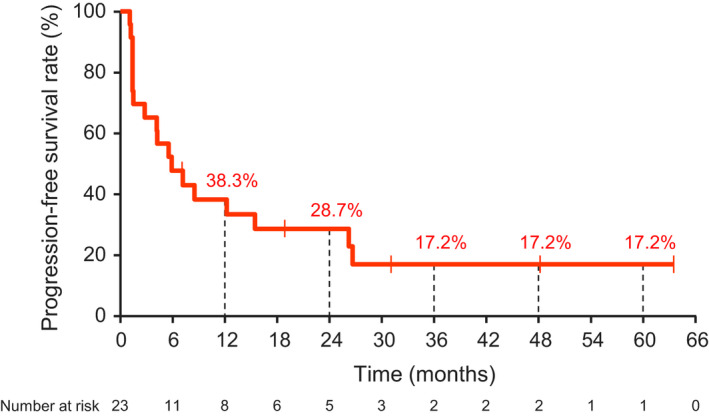
Kaplan–Meier curve of progression‐free survival (central assessment)

The median OS was 32.9 months, and the 4‐ and 5‐year survival rates were 30.4% and 26.1%, respectively (Figure 1). The median PFS was 5.9 months, and the 4‐ and 5‐year PFS rates were both 17.2% (Figure 2). The ORR was 34.8% (90% CI, 20.8–51.9). The DCR was 65.2% (90% CI, 48.1–79.2). There were no new cases of partial response (PR) or CR after 3 years, and the ORR and DCR were similar to those reported at 3 years.

**FIGURE 3:**
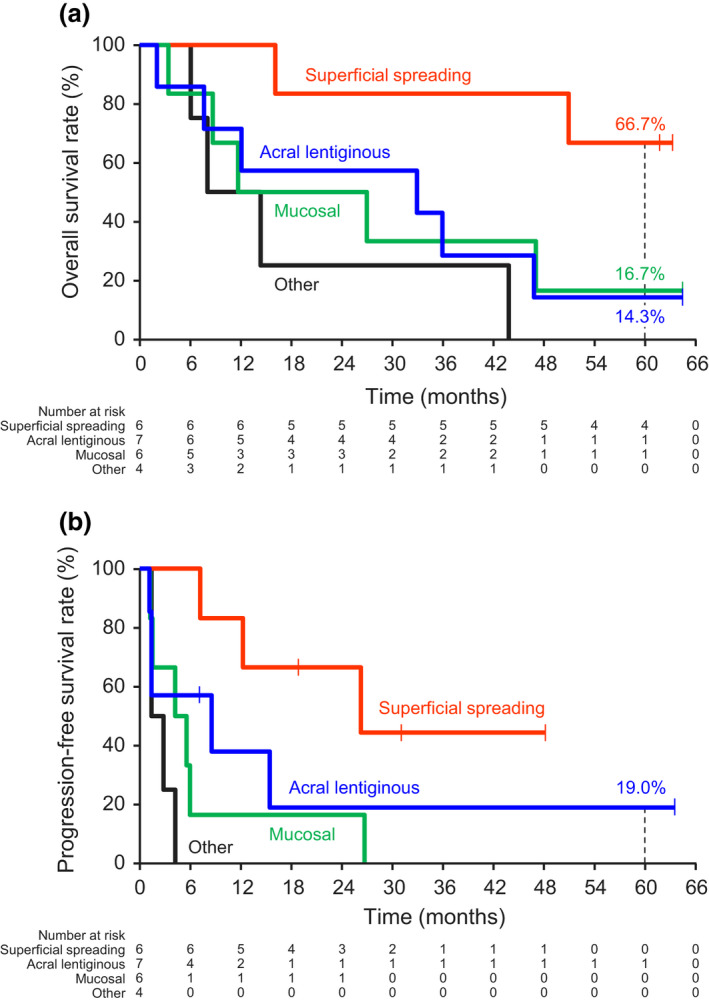
Kaplan–Meier curves of (a) overall survival in subgroups and of (b) progression‐free survival (central assessment) in subgroups by melanoma type

Among patients with superficial spreading melanoma, the median OS was not reached, and the 5‐year OS rate was 66.7%. The respective median OS and 5‐year OS rates were 32.9 months and 14.3% for acral lentiginous type, 19.3 months and 16.7% for mucosal type, and 11.1 months and 0% for other types of melanoma (Figure 3a). The 5‐year OS rate was higher for superficial spreading type than for mucosal and acral lentiginous types. The same tendency was observed for PFS (Figure 3b). The median OS for patients with *BRAF* mutation was not calculable because 50% of patients were still alive by the data cut‐off; that for patients without *BRAF* mutation was 26.9 months (Figure S1a). The corresponding PFS data by *BRAF* mutation status are shown in Figure S1b.

**FIGURE 4:**
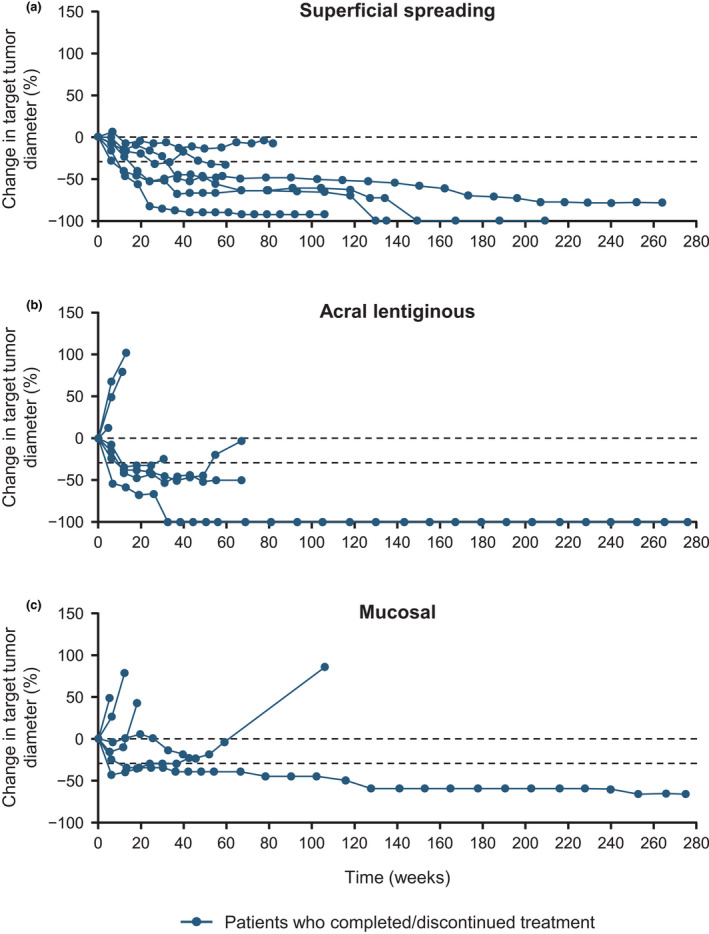
Percent change in target tumor diameter over time (investigator’s assessment at the study site) in subgroups by (a) superficial spreading melanoma, (b) acral lentiginous melanoma, and (c) mucosal melanoma

**FIGURE 5:**
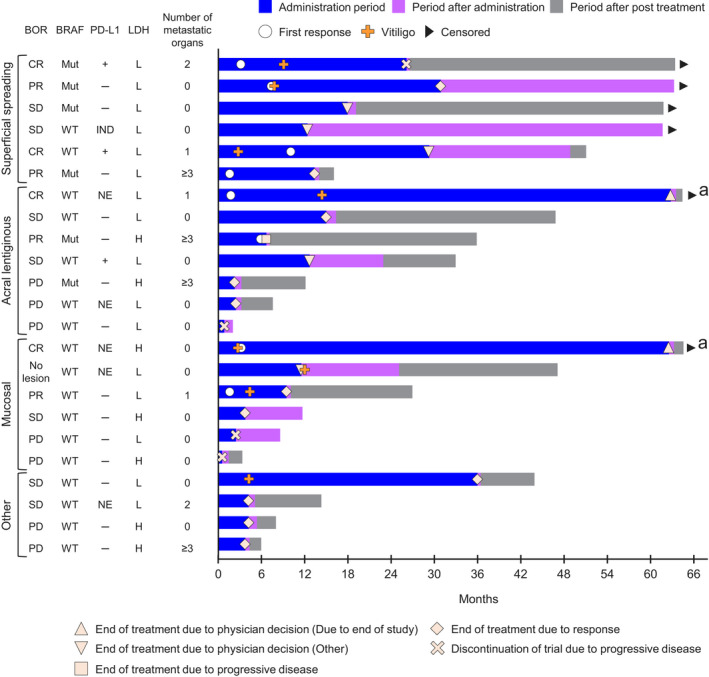
Swimmer plot showing individual treatment progress by melanoma type. ^a^Although nivolumab treatment was discontinued at the end of the study, two patients continued to receive post‐treatment nivolumab. PD‐L1+ indicates positive (≥1%) and PD‐L1− indicates negative (<1%). L indicates LDH ≤upper limit of normal and H indicates LDH >upper limit of normal. BOR, best overall response; CR, complete response; H, high; IND, indeterminable; L, low; LDH, lactate dehydrogenase; Mut, *BRAF* mutant; NE, not evaluable; PD, progressive disease; PD‐L1, programmed death ligand 1; PR, partial response; SD, stable disease; WT, *BRAF* wild‐type

Changes in tumor diameter for individual patients by melanoma subtype are shown in Figure 4, and long‐term responses were observed in all three of the main melanoma subtypes. Figure 5 shows the individual treatment response and treatment progress by melanoma subtype. Five years after the start of nivolumab treatment, there were six survivors. Among these 5‐year survivors, four had the superficial spreading type (66.7%), one had the acral lentiginous type (16.7%), and one had the mucosal type (16.7%). In the 17 patients who did not survive for 5 years (non‐5‐year survivors), two had the superficial spreading type (11.8%), six had the acral lentiginous type (35.3%), and five had the mucosal type (29.4%). In addition, among 5‐year survivors, the BOR was CR in 50.0% (3/6), PR in 16.7% (1/6), and stable disease in 33.3% (2/6). The corresponding values in non‐5‐year survivors were 5.9% (1/17), 17.6% (3/17), and 29.4% (5/17), respectively. Among the 5‐year survivors, 83.3% (5/6 patients) had lactate dehydrogenase (LDH) levels below the upper limit of normal, 66.7% (4/6 patients) had vitiligo, and 33.3% (2/6 patients) had organ metastases. In non‐5‐year survivors, 64.7% (11/17 patients) had LDH levels below the upper limit of normal, 23.5% (4/17 patients) had vitiligo, and 41.2% (7/17 patients) had organ metastases.

Table S1 summarizes the results of the subgroup analysis of clinical efficacy measures, according to melanoma type. The ORR and DCR were 66.7% and 100%, 28.6% and 57.1%, 33.3% and 50.0%, and 0% and 50.0% for superficial spreading, acral lentiginous, mucosal, and other melanoma types, respectively. The median OS and PFS were not reached and 26.2 months, 32.9 and 8.5 months, 19.3 and 4.9 months, and 11.1 and 2.1 months for superficial spreading, acral lentiginous, mucosal, and other melanoma types, respectively. In this subgroup analysis, the patients with superficial spreading melanoma type tended to have higher OS, ORR, DCR, and PFS than patients with mucosal, acral lentiginous, and other melanoma types.

### 3.3 Safety end‐points

The treatment‐related AE that occurred between the 3‐ and 5‐year follow‐up were anemia (grade 2), white blood cell count decreased (grade 2), and psoriasiform dermatitis (grade 1) in one patient each. No new grade 3 or higher treatment‐related AE occurred during this period (data not shown). In addition, the safety profile during this period was similar to that described in the previous report, and no new safety signals were observed (Table S2).

## 4 DISCUSSION

This analysis of the ONO‐4538‐08 study investigated the long‐term efficacy (5‐year OS and PFS) and safety of nivolumab for Japanese patients with untreated advanced or recurrent malignant melanoma. The 5‐year OS rate was 26.1%, indicating that first‐line treatment with nivolumab in Japanese patients with unresectable or metastatic melanoma resulted in long‐term survival for some patients. However, the OS rate reported in the present study was lower than the rates reported in the CheckMate 066^7^, ^8^ and 067^9^, ^10^ trials (34% and 44%, respectively). It has been reported that the proportion of patients with acral lentiginous type and mucosal type melanoma is higher in Japan and other East Asian countries compared with the rest of the world.^11^, ^12^, ^13^, ^14^ In this study, acral lentiginous type was the most common melanoma type (29.2%), followed by mucosal type (25.0%). In contrast, a study in Caucasians reported that the proportion of acral lentiginous and mucosal types was 3.0% and 6.9%, respectively.^15^ In our analysis, the 5‐year OS rate was 66.7% in patients with superficial spreading melanoma, and this rate was higher than that in patients with acral lentiginous (14.3%) and mucosal (16.7%) types. The same tendency was observed for PFS. These results were also similar to those reported in the 3‐year analysis.^4^ Poor prognosis in Japanese patients with mucosal and acral lentiginous types has also been reported in another study in which patients were administrated anti‐PD‐1 antibodies.^16^ Overall, these findings suggest that the melanoma type may affect prognosis in patients treated with anti‐PD‐1 antibodies. One potential reason for these differences may be related to the tumor mutational burden (TMB). Reportedly, the TMB is low in the acral lentiginous and mucosal types compared with the superficial spreading type.^17^ In cancer cells with a high TMB, the immune system is thought to more easily recognize the tumor due to increased neoantigen levels. This might explain the variable response to nivolumab between the superficial spreading type and acral lentiginous and mucosal types.

The incidence of grade 3 or more treatment‐related AE in our analysis was 12.5%, and no new safety signals were identified when compared with the initial (18‐month) and 3‐year analyses.^4^, ^5^ The ONO‐4538‐17 study^18^ reported that 76.7% of patients in the nivolumab + ipilimumab group had grade 3–4 treatment‐related AE compared with 12.5% of patients in the nivolumab group in this study. Numerically, patients receiving nivolumab + ipilimumab combination therapy had a high incidence of grade 3–4 treatment‐related AE, a tendency that was also reported in the nivolumab + ipilimumab combination therapy arm of the CheckMate 067 trial.^9^, ^10^ Thus, the incidence of grade 3 or more treatment‐related AE should be considered when choosing treatment based on melanoma type. However, because of the small sample size and limited number of reports, further studies are needed to clarify the relationship between immune checkpoint inhibitor efficacy and disease type.

There were six survivors at 5 years after the start of nivolumab treatment in the present study. Among these six, the BOR was variable (CR in 50.0% [3/6], PR in 16.7% [1/6], and stable disease in 33.3% [2/6]). However, the survivors seemed to have some characteristics in common, including a superficial spreading disease type (67%, 4/6 patients), a serum LDH level below the upper limit of normal (83%, 5/6 patients), and vitiligo (67%, 4/6 patients). The values of these factors were numerically higher among 5‐year survivors compared with non‐5‐year survivors, and these relationships were similar to those observed in previous reports.^6^, ^19^, ^20^, ^21^, ^22^, ^23^, ^24^ Of note, the relationship between the onset of vitiligo and the efficacy of nivolumab is somewhat unclear. Two patients among the 5‐year survivors did not develop vitiligo during nivolumab treatment and two of the four 5‐year survivors who developed vitiligo experienced these events after the initial response. Therefore, further assessment of this relationship is needed.

The main limitations of this study have been previously described,^4^, ^5^ and consist mainly of the open‐label design, the lack of a comparator, and the small sample size. The results presented here should be confirmed in a large‐scale clinical trial.

In conclusion, first‐line treatment with nivolumab in Japanese patients with unresectable or metastatic melanoma resulted in long‐term survival for some patients. No new safety signals were reported in the studied population.

## CONFLICT OF INTEREST

H. Uhara has received honoraria from Ono Pharmaceutical and Novartis, manuscript fees from Ono Pharmaceutical, and research funding from Ono Pharmaceutical, Maruho, Taiho Pharmaceutical, and Torii. Y.F. has received honoraria from Novartis and research funding from Eisai. T.T. has received honoraria from Ono Pharmaceutical, MSD, Novartis, and Bristol‐Myers Squibb. S.F. has received grants from Ono Pharmaceutical. H.M. has received honoraria from Ono Pharmaceutical and grants from Ono Pharmaceutical and Bristol‐Myers Squibb. M.H. is an employee of Ono Pharmaceutical. N.Y. has received honoraria from Ono Pharmaceutical, Novartis, Bristol‐Myers Squibb, MSD, Chugai, Takeda, and Merck, and research funding from Ono Pharmaceutical, Novartis, MSD, Bristol‐Myers Squibb, Merck, Takara Bio, and Sysmex. Y.K., J.U., M.O., and H. Uchi have no conflict of interest related to this research.

## Supporting information

Table S1
Table S2
Figure S1
