# Supplementary material for: Five‐year survival with nivolumab in previously untreated Japanese patients with advanced or recurrent malignant melanoma
Source: J Dermatol. 2021 Mar 14;48(5):592–9. doi: 10.1111/1346-8138.15804 (PMC8252056; doi:10.1111/1346-8138.15804)
Supplement: Supplementary file 1 — Table S1 Table S2 Figure S1 [file JDE-48-592-s001.pdf]

Table S1. Summary of subgroup analysis according to the sum of tumor diameter and melanoma type at baseline

	**All patients (n)**	**ORR n (%) [90%CI]**	**DCR n (%) [90%CI]**	**OS Median (months)**	**PFS Median (months)**
Melanoma type					
Superficial spreading	6	4 (66.7) [34.7–88.3]	6 (100) [68.9–100.0]	NR	26.2
Acral lentiginous	7	2 (28.6) [10.0–59.1]	4 (57.1) [28.9–81.4]	32.9	8.5
Mucosal	6	2 (33.3) [11.7–65.3]	3 (50.0) [22.1–77.9]	19.3	4.9
Other	4	0 (0.0) [0.0–40.3]	2 (50.0) [18.2–81.8]	11.1	2.1

CI, confidence interval; DCR, disease control rate; NR, not reached; ORR, overall response rate; OS, overall survival; PFS, progression-free survival

Table S2. Incidence of treatment-related adverse events observed in ≥10% of all patients

Incidence, n (%)	All patients (N = 24)	
	All grade	Grade ≥3
Overall	20 (83.3)	3 (12.5)
Vitiligo	9 (37.5)	0
Hypothyroidism	6 (25.0)	0
Malaise	6 (25.0)	0
Pruritus	6 (25.0)	0
Nausea	3 (12.5)	0
Weight decreased	3 (12.5)	0
White blood cell count decreased	3 (12.5)	0
Appetite decreased	3 (12.5)	0
Arthralgia	3 (12.5)	0
Rash maculo-papular	3 (12.5)	0

The adverse event terms reported by physicians were coded using the Japanese Medical Dictionary for Regulatory Activities (MedDRA/J) version 22.1J and graded according to the Common Terminology Criteria for Adverse Events (CTCAE; version 4.0).

This table summarizes adverse events that occurred from the start of the treatment period to day 28, or to the start day of subsequent treatment following the last administration of the study drug, whichever occurred first. Adverse drug reactions were defined as adverse events whose causal relationship with the study drug cannot be ruled out.

Fig. S1. Kaplan–Meier curves of (a) overall survival by BRAF genotype and of (b) progression-free survival by BRAF genotype (central assessment)

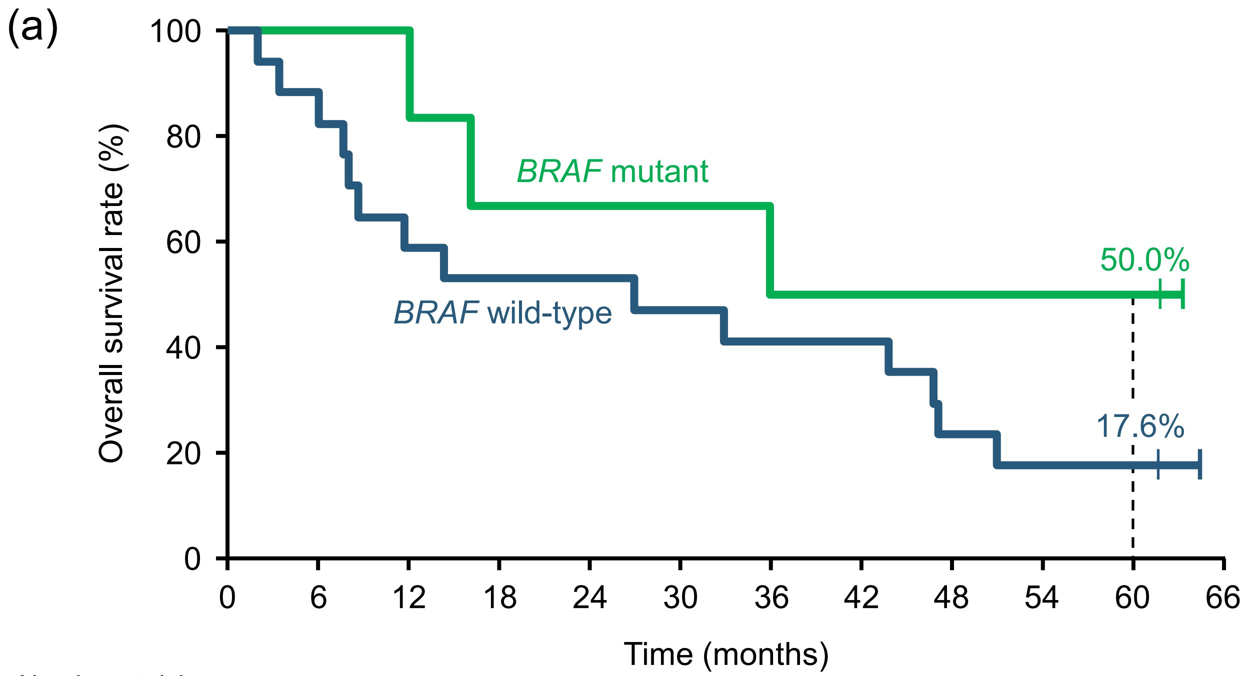

Number at risk												
Wild-type	17	14	10	9	9	8	7	7	4	3	3	0
Mutant	6	6	6	4	4	4	3	3	3	3	3	0

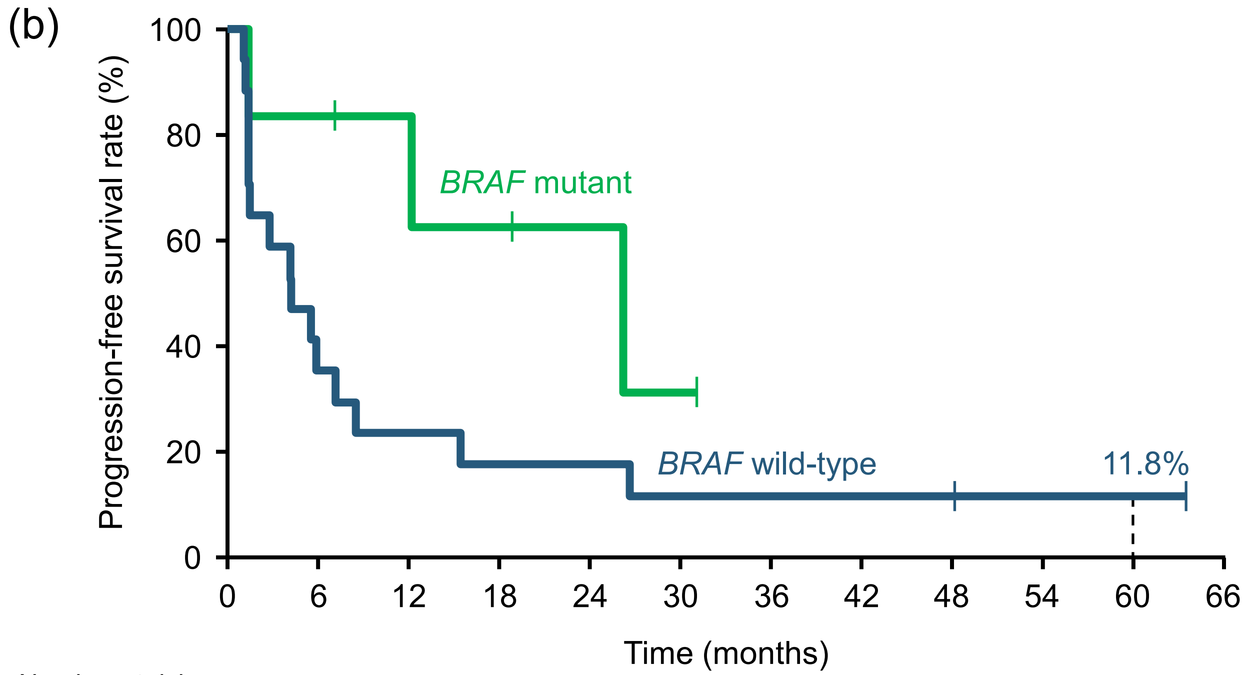

Number at risk												
Wild-type	17	6	4	3	3	2	2	2	2	1	1	0
Mutant	6	5	4	3	2	1	0	0	0	0	0	0
